# Information-geometric optimization of transient dynamics in gene regulatory networks

**DOI:** 10.1098/rsos.261278

**Published:** 2026-10-21

**Authors:** Alessandro Bravetti, Miguel Ángel García Ariza, Pablo Padilla, José Roberto Romero-Arias

**Affiliations:** ^1^ School of Science and Technology, University of Camerino, Camerino, Italy; ^2^ IIMAS, Universidad Nacional Autónoma de México, Mexico City, Mexico

**Keywords:** signalling networks, information theory, noise, optimization

## Abstract

The dynamics of gene regulatory networks (GRNs) is governed by the interaction between deterministic biochemical reactions and molecular noise. To understand how GRNs process information during cell state transitions, we study stochastic dynamics derived from a Boolean network model via its representation on the parameter space of Gaussian distributions, equipped with the Fisher information metric. Restricting our analysis to transitions towards a single attractor, this reformulation reveals that dynamics along the gradient flows of the Kullback–Leibler (KL) divergence requires isotropic decay rates across all nodes, and that the noise intensity quantitatively determines the potential differentiation between the initial and final states. Furthermore, we show that the Fisher geodesics, previously identified with minimal biological cost, would require the suppression of noise and lead to deterministic dynamics that lacks biological relevance. This suggests that some departure from minimal cost is necessary for cell differentiation. Our approach frames noise and decay rates as fundamental control parameters, providing a geometric principle for the analysis and design of synthetic networks.

## 1. Introduction

In biochemistry, a useful way to think about molecular relationships is in terms of interaction networks. These provide a simplified and powerful representation of cellular systems, capturing relationships between metabolites, proteins and mRNAs. Among these, gene regulatory networks (GRNs) stand out as key models for understanding gene expression. They are depicted as directed graphs, the dynamics of which can be described in different ways, including logical formalisms and chemical kinetics.

To understand how GRNs process information, we need frameworks that integrate biological processes across multiple scales. Research has shown that morphogenetic and topological constraints play a central role in regulatory mechanisms, transcriptional pathways and overall system robustness in biological systems [1–9]. Recently, we have seen remarkable advances in constructing GRNs and characterizing their topological features [9–12]. However, a dynamical description of information transfer has often been overlooked, prompting a re-evaluation of theoretical approaches to systems biology [13–16]. This gap motivates interdisciplinary work to develop a mathematical language to quantify the flow, storage and transformation of information within biological networks across multiple scales where fundamental life processes occur [17–21].

Information theory provides powerful tools for these purposes [22,23], with applications in various fields, including neuroscience [24–26], biochemical networks [27,28], statistical physics [29–31], music [32] and artificial intelligence [33,34]. Particularly relevant is *information geometry* [35], which offers a geometric approach to characterize the dynamics of complex systems far from equilibrium [36,37]. This framework provides metrics derived from information theory that offer new insights for analysing networks [38–40]. Specifically in the context of GRNs, these methods can reveal how network topologies shape signal pathway fidelity, system robustness and phenotypic complexity [41–43].

The aim of this paper is to understand how GRNs process information along stochastic dynamics described by the Fokker–Planck (FP) equation. This approach enables us to quantify the Waddington landscape [44] close to an attractor, and the biological pathways [1,45] for development and differentiation, providing a geometric perspective on cellular decision-making processes. Although existing studies have addressed non-stationary dynamics in GRNs [46–48], a geometric characterization of relaxation processes of GRNs remains relatively unexplored. Here, we address this problem by applying information geometry to GRNs, translating the stochastic dynamics into a deterministic flow on a finite-dimensional space equipped with a metric tensor, following a procedure similar to the one used by Obata *et al.* [49] in the context of Ornstein–Uhlenbeck processes. This allows us to characterize the gradient descents of the Kullback–Leibler (KL) divergence as geodesics, while revealing how noise and decay rates drive the network dynamics along these trajectories. Our framework also allows us to show that metric geodesics (minimizing statistical distance) require the suppression of noise and appear to have limited biological relevance.

To formalize these ideas, we first explain how we represent the stochastic dynamics of the network with a deterministic framework. We then introduce the KL divergence and the Fisher metrics, which allow us to define the gradient flow of the divergence and compare it with the deterministic representation of Langevin dynamics. Next, we study the evolution of networks that occur along geodesics, including those that minimize the statistical distance between states. Finally, we conclude with remarks and perspectives for future work, supplemented by detailed calculations and deeper geometric explanations in the appendices.

## 2. Stochastic gene-network model

There are two common approaches to model GRNs: continuous descriptions based on ordinary differential equations arising from chemical kinetics, and discrete descriptions in which logical rules determine the dynamics. Here, we start from a Boolean network of N nodes and derive a continuous stochastic dynamics following the methodology introduced by Villarreal *et al.* [1]. The state of the ith node at time t+1 is given by $q_{i}(t+1)=w_{i}(q(t))$, where $w_{i}$ are logical propositions and $q=(q_{1},\ldots ,q_{N})$ are dichotomic variables representing the expression level of each gene (0=inactive, 1=active).

Following [1], to transform this discrete system into a continuous one, we employ fuzzy propositional calculus (Zadeh's rules), which replaces logical operations by continuous functions on the interval [0,1]. In this way, each Boolean input function $w_{i}$ is turned into a continuous function $w_{i}(q)$ that expresses how the regulators of node i interact. The stochastic dynamics of the continuous expression level $q_{i}$ is governed by the Langevin equation [1],

(2.1) $$\frac{dq_{i}}{dt}=\Theta [w_{i}(q)]-\alpha_{i}q_{i}+\xi_{i},$$

where $\Theta [w_{i}]$ is a sigmoidal activation function (with threshold $w_{i}^{\text{thr}}$ and saturation rate b, so that $\Theta [w_{i}]$ tends to the Heaviside step function for b≫1), $\alpha_{i}$ is the relaxation (degradation) rate of the gene product and $\xi_{i}$ is Gaussian white noise with zero mean and time correlation $\left\langle \xi_{i}(t)\xi_{j}(t^{\prime })\rangle =Q\delta_{ij}\delta (t-t^{\prime }\right)$, which captures the stochastic nature of the underlying biochemical reactions.

## 3. Deterministic representation of the stochastic dynamics

As the network evolves under equation (2.1), the probability distribution of gene-expression states p(q;t) evolves according to the associated FP equation $\partial_{t}p(q;t)=-\nabla_{q}\cdot J$, where the probability current J has components [1]

(3.1) $$J_{i}=\{\Theta [w_{i}(q)]-\alpha_{i}q_{i}\}p(q;t)-\frac{1}{2}Q\frac{\partial p}{\partial q_{i}}.$$

In the adiabatic regime, where the relaxation dynamics of the variables is much faster than the changes of the input functions $\Theta [w_{i}]$, the drift term can be linearized around the fixed-point attractor. The resulting FP equation then has linear drift coefficients and a constant diffusion matrix, whose solutions are well known to be Gaussian distributions [50]. Villarreal *et al.* [1] apply this result to obtain

(3.2) $$p(q,t)=\prod_{i=1}^{N}\left[\frac{1}{\sqrt{a_{i}(t)\pi }}\exp\left\{\frac{(q_{i}-\mu_{i}(t){)}^{2}}{a_{i}(t)}\right\}\right],$$

where the parameters $\mu_{i}(t)$ and $a_{i}(t)$ represent, respectively, the mean and variance of the state of the ith node of the network and evolve according to

(3.3a)μi(t)=μi(0)e−αit+qi∗(1−e−αit)(3.3b)and ai(t)=ai(0)e−2αit+ai∗(1−e−2αit).

This description is valid under two approximations. First, we work in the adiabatic regime, where the relaxation rates $\alpha_{i}$ are sufficiently large that the input functions $\Theta [w_{i}]$ may be treated as constant. Second, we assume that the network states remain within the basin of attraction of a single fixed-point attractor $q_{i}^{*}$ [44], characterized by $q_{i}^{*}=\Theta [w_{i}(\langle q\rangle )]/\alpha_{i}$ and $a_{i}^{*}=Q/\alpha_{i}$. Restricting the analysis to a single basin is an important limitation of our work, but we regard it as a first step towards a geometric understanding of how information evolves within GRNs during cell differentiation.

**Figure 1.**
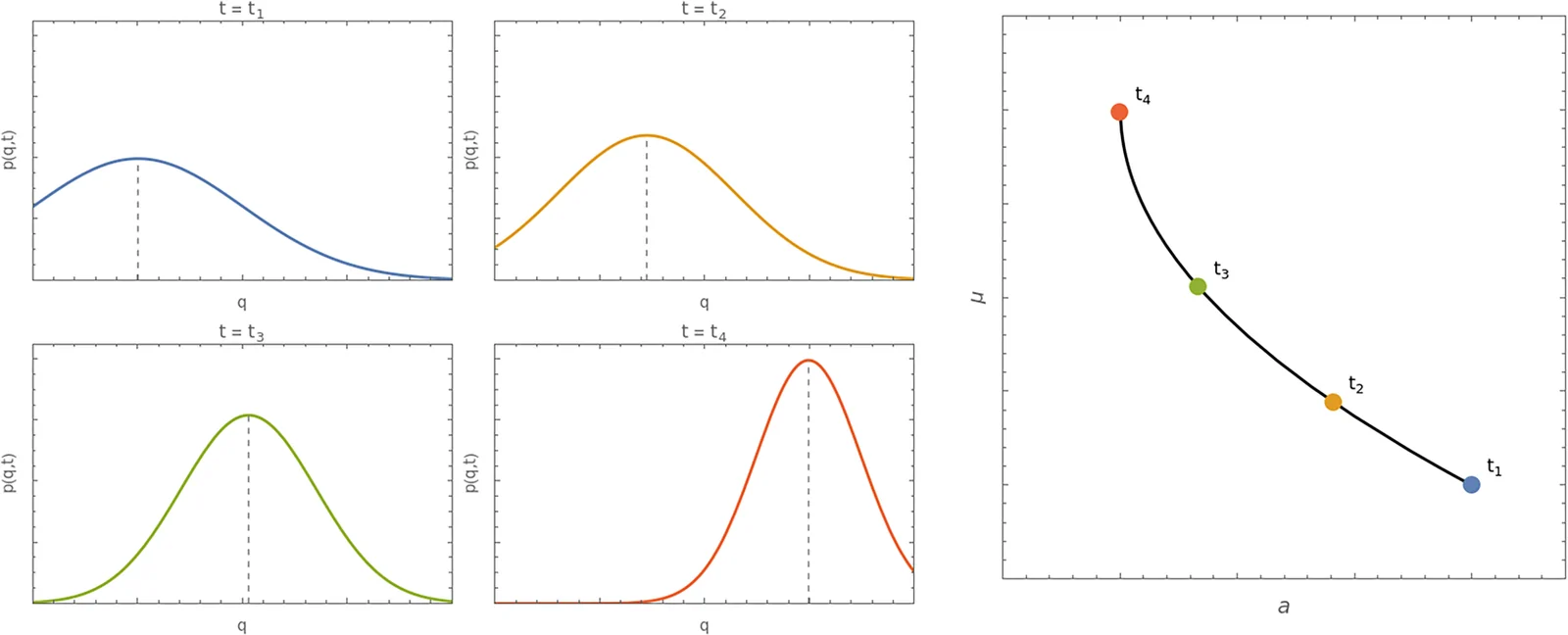
This illustrates how we represent the FP dynamics of the network with a deterministic one. The left-hand panels depicts the evolution of p(q;t) in time for one node of the network. Each one of these distributions corresponds to a point in parameter space. Thus, the solution to the corresponding FP equation can be regarded as a curve on this finite-dimensional space.

The solution of the FP equation translates the stochastic network dynamics into deterministic trajectories in a finite-dimensional parameter space spanned by $\left\{(\mu_{i},a_{i})\right\}$ (figure 1). The corresponding flow is obtained by differentiating equations (3.3) (see appendix A for explicit calculations):

(3.4a)μ˙i=−αi(μi−qi∗)(3.4b)and a˙i=−2αi(ai−Qαi).

This reduction of the stochastic dynamics to a deterministic flow on a finite-dimensional parameter space is the essential first step of our geometric analysis. Although equations (3.4) are derived for the specific model given by equation (2.1), the overall procedure is quite general: given a parametric family of probability distributions that solves the associated FP equation, the dynamics of the parameters defines a flow on a finite-dimensional space. Its geometric properties can then be studied with the tools of information geometry. The practical feasibility of this approach depends on the availability of an analytical or approximate solution of the FP equation. Obata *et al.* [49] apply this exact idea to Ornstein–Uhlenbeck processes.

We stress that this translation from stochastic to deterministic finite-dimensional dynamics is fundamentally different from the gradient-flow formulation of the FP equation in the space of probability distributions recently discussed by Ito [51]. There, *every* FP dynamics is shown to be a gradient flow with respect to the $L^{2}$-Wasserstein metric [52]. Here, by contrast, we restrict the dynamics to a prescribed parametric family and ask under which conditions the resulting flow in the parameter space coincides with the natural gradient of a divergence *with respect to the Fisher information metric*. The two descriptions are complementary: the former concerns the infinite-dimensional space of probability densities, whereas the latter concerns a finite-dimensional parameter space. The interplay between these two geometric structures is currently a very active line of research [53–57]

Returning to GRNs; as the network converges to a steady state, its current state grows increasingly ‘similar’ to this final attractor in terms of their information content. This ‘similarity’ is quantified by the *KL divergence*. Divergences are asymmetric ‘distance-like’ measures between distributions. Unlike proper distances, they do not obey the triangle inequality in general, but are non-negative and vanish only when the two distributions coincide. In particular, the KL divergence is defined as

(3.5) $$D_{\text{KL}}(P_{1}(q)||P_{2}(q)):=\int_{Q}P_{1}(q)\log\left(\frac{P_{1}(q)}{P_{2}(q)}\right)dq,$$

where integration runs over all possible network states q∈Q, which we assume continuous.

Since the network gains certainty about the steady state as it evolves in time, it is natural to wonder about dynamics that maximizes the rate at which this information gain occurs. Geometrically, this amounts to asking when equation (3.4) acts as the gradient flow of the KL divergence.

To consider the gradient descent of the KL divergence, we need to pin down exactly what we mean by *gradient*, for which two common notions exist. First, we have the so-called *vanilla gradient* (vector of partial derivatives), which depends on how we parametrize the distributions. This is problematic because if we reparametrize, the new steepest descent direction might not coincide with that given by the natural parameters $\left(\mu_{i},a_{i}\right)$. The second notion, the *natural gradient*, avoids this pitfall, since it is coordinate-independent. Although its definition requires a metric tensor, this is not a difficulty in this case, since the KL divergence provides us with one [35].

To show this, we observe that the KL divergence induces a divergence on the space spanned by the parameters η of *any* family of distributions:

$$\begin{array}{r} D(\eta_{1}||\eta_{2}):=D_{\text{KL}}(p(q;\eta_{1})||p(q;\eta_{2})). \end{array}$$

This is well defined if we assume that the identification η↦p(q;η) is one-to-one. Furthermore, as we show in appendix A, for nearby parameters,

(3.6) $$D(\eta ||\eta +\delta \eta )\approx -\frac{1}{2}E_{\eta }\left[\frac{\partial^{2}\ell_{q}}{\partial \eta^{i}\partial \eta^{j}}\right]\delta \eta^{i}\delta \eta^{j},$$

with $\ell_{q}(\eta ):=\log p(q;\eta )$ denoting the log-likelihood and $E_{\eta }[\cdot ]$ meaning the expectation with respect to p(q;η). Above, and in what follows, we use Einstein's summation convention over repeated indices. We show that, in particular, for the family of distributions defined by equation (3.2), where $\eta =(\mu_{i},a_{i})$ (see appendix B),

$$\begin{array}{r} \begin{array}{rl} E_{\eta }\left[\frac{\partial^{2}\ell_{q}}{\partial \mu_{i}\partial \mu_{j}}(q;\eta )\right] & =-\frac{2}{a_{i}}\delta_{ij},\\ E_{\eta }\left[\frac{\partial^{2}\ell_{q}}{\partial \mu_{i}\partial a_{j}}(q;\eta )\right] & =0\\ \text{and }E_{\eta }\left[\frac{\partial^{2}\ell_{q}}{\partial a_{i}\partial a_{j}}(q;\eta )\right] & =-\frac{\delta_{ij}}{2a_{i}^{2}}. \end{array} \end{array}$$

With $a_{i}<0$, the matrix with entries $g_{ij}:=-E_{\eta }[\partial_{i}\partial_{j}\ell_{q}]$ is positive definite, and thus defines a metric tensor on the parameter space, called *Fisher metrics*. We may use it to compute the gradient flow of the KL divergence in the next section.

Note that $g_{ij}$ (above) yields a positive definite matrix for *any* parametric family of distributions [35]. In other words, the parameter space of every such family is a Riemannian space with Fisher metrics. This is one of the three ingredients of *information geometry*.

**Figure 2.**
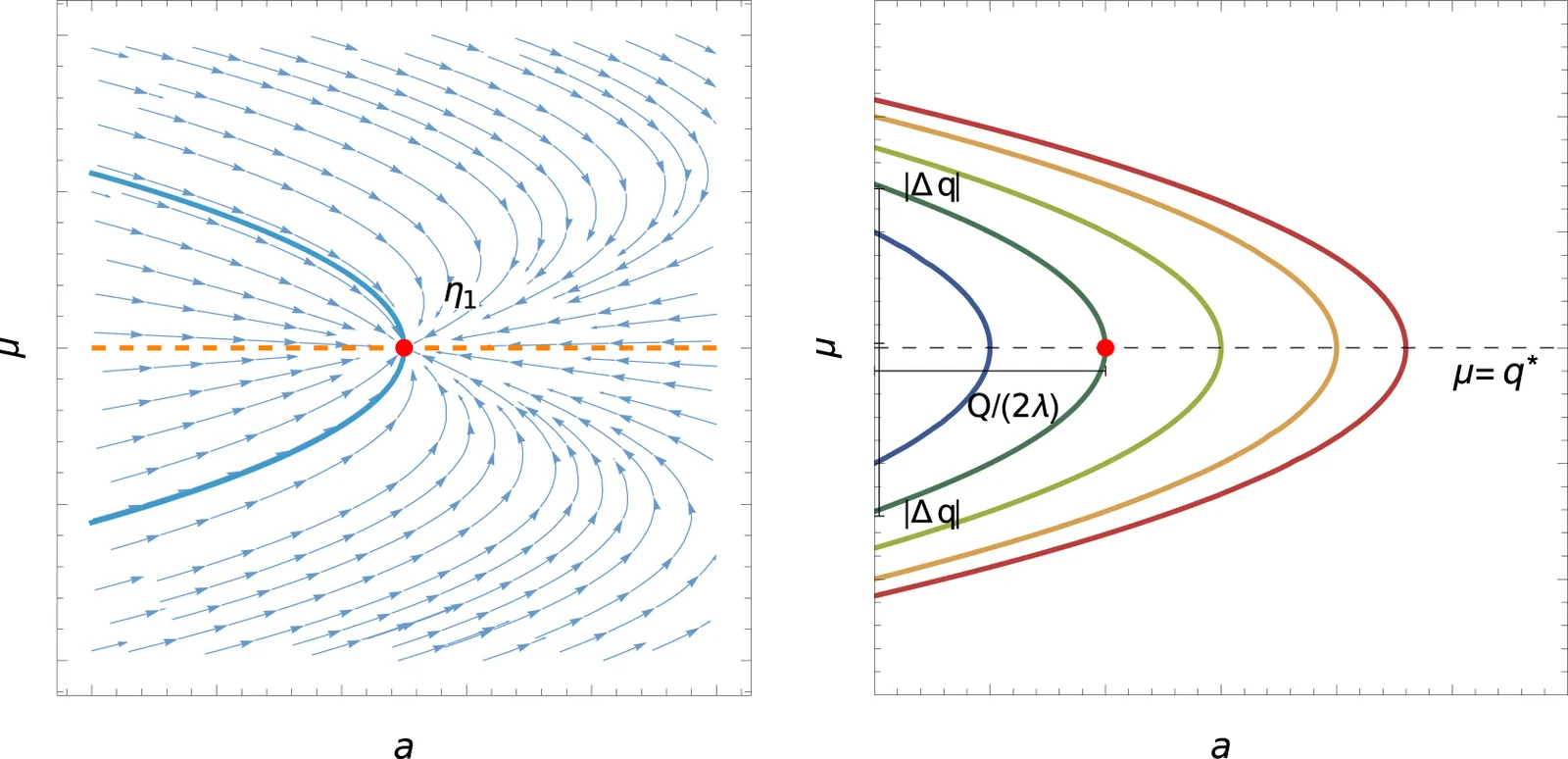
Left-hand panel: the gradient flow of $D_{1}$ on an $a_{i}$–$\mu_{i}$ plane, for some fixed i. The solid curve and the dashed line are the only trajectories of the dynamics that also correspond to the FP dynamics of the node. Right-hand panel: along a gradient descent curve of $D_{1}$ (equation (4.4)), $a_{i}^{*}=Q/(2\lambda )$, and thus greater noise (blue to red) allows for a greater differentiation between the initial and the final states of the network, $\left|\Delta q_{i}|:=|\mu_{i}(0)-q_{i}^{*}\right|$.

## 4. Dynamics of fastest learning about the steady state

For the Gaussian family in equation (3.2), the KL divergence is (see the supplementary material)

(4.1) $$D((\mu_{1},a_{1})||(\mu_{2},a_{2}))=\sum_{i=1}^{N}\left\{\frac{1}{2}\left[\frac{{a_{i}}_{1}}{{a_{i}}_{2}}-\log\left(\frac{{a_{i}}_{1}}{{a_{i}}_{2}}\right)-1\right]+\frac{({\mu_{i}}_{1}-{\mu_{i}}_{2}{)}^{2}}{{a_{i}}_{2}}\right\}.$$

Fix $\eta_{1}:=\left(q_{i}^{*},a_{i}^{*}\right)$. To compute the gradient of $D(\eta_{1}||\cdot )=D_{1}$, we apply the inverse Fisher metrics $g^{ij}$ to $\partial_{j}D_{1}$, obtaining the following *Riemannian* gradient flow converging to $\eta_{1}$ [58]:

(4.2a)μ˙i=−λ(μi−qi∗)(4.2b)and a˙i=−λ[ai−ai∗−2(μi−qi∗)2],

where λ<0. This flow is represented in the left-hand panel of figure 2 for one node of the network.

Comparing this with equation (3.4), the network evolves along the gradient flow of $D_{1}$ if

(4.3a)−αi(μi−qi∗)=−λ(μi−qi∗)(4.3b)and −2αi(ai−ai∗)=−λ[ai−ai∗−2(μi−qi∗)2].

These equations constrain the decay rates $\alpha_{i}$ and steady-state values $\left(q_{i}^{*},a_{i}^{*}\right)$. To see how, we consider three separate cases. (i) First, observe that fixed variances ($a_{i}(t)\equiv a_{i}^{*}$) imply constant means. So, maximizing the rate at which $D_{1}$ decreases requires non-constant variances. (ii) Second, if the means are fixed ($\mu_{i}(t)\equiv q_{i}^{*}$), all $a_{i}$ must decay uniformly at a rate $\alpha_{i}\equiv \lambda /2$, as follows from equation (4.3*b*). (iii) Third, when the $\mu_{i}$ are non-constant, they also decay uniformly, but at a rate $\alpha_{i}\equiv \lambda$ along the curve

(4.4) $$a_{i}-a_{i}^{*}=-2{\left(\mu_{i}-q_{i}^{*}\right)}^{2}.$$

For one node in the network, this curve corresponds to the solid parabola in the left-hand panel of figure 2. In this case, the variances also decay uniformly. Furthermore, if the initial $a_{i}(0)$ is close to zero, the $a_{i}(t)$ correspond to the mean square fluctuations of $q_{i}$, i.e. $a_{i}=\langle q_{i}^{2}(t)\rangle -\langle q_{i}(t)\rangle^{2}$ [1]. Recalling that $a_{i}^{*}=Q/\alpha_{i}$, this leads to a relationship between the noise intensity and the parameters of the dynamics that resembles a fluctuation dissipation theorem, where the noise intensity Q is approximately proportional to the product of the decay rate λ (analogous to a damping constant) and the square of the transition distance:

(4.5) $$Q\approx 2\lambda {\left(\mu_{i}(0)-q_{i}^{*}\right)}^{2},$$

as follows after substituting equation (3.3) into the relaxation curve equation (4.4). This result suggests that in the regime of small uncertainties, a larger differentiation is inherently tied to higher levels of stochastic fluctuations in the dynamics. Note that this result is observed in various genetic processes such as transcription, coordinated gene expression, signalling pathways and cell fate [12,15,16,18,59]. In the right-hand panel of figure 2, we illustrate how noise allows for a greater difference between the initial and final expression levels of one node.

To close this section, we recall that the function $D_{1}$ measures how inefficient it is, in terms of information, to code samples of the steady-state distribution $p(q;\eta_{1})$ using a code optimized for the current distribution p(q;t) [22]. In other words, it quantifies the amount of information the network still lacks about equilibrium [60]. Our first finding is that, to obtain this missing information in the fastest manner, all variances must decay uniformly. In informal terms, the system *learns* the steady-state distribution and gains certainty about its fate the fastest in this situation.

A recent result by Crooks [61] shows that $D_{1}$ also admits a precise thermodynamic interpretation: it is the excess free energy of a thermodynamically dual ensemble, where the roles of entropy and energy are interchanged [61]. Its gradient then maximizes the rate at which this dual free energy is dissipated, complementing the information-theoretic picture described above.

## 5. Dynamics of fastest forgetting about the initial state

Divergences are non-symmetric; while $D_{1}$ quantifies the amount of knowledge the network still lacks about the steady state [60], its dual $D(\cdot ||\eta_{1})=:D_{1}^{*}$ measures how inefficient it is to describe the current state p(t) using a code optimized for equilibrium [22]. A decrease in $D_{1}^{*}$ therefore implies that the system is progressively *forgetting* its initial condition, and the gradient flow of $D_{1}^{*}$ then maximizes the rate at which this forgetting occurs (Burgholzer provides a similar interpretation of this quantity for systems returning to equilibrium after being kicked out of it [62].) This flow is given by

(5.1a)μ˙=−λaiαiQ(μi−qi∗)(5.1b)and a˙i=−λaiαiQ(ai−Qαi).

See appendix B for details. In figure 3, we show the gradient flow of the dual divergence for one node. The solid line represents the only solution to the FP equation along this flow, which we obtain by matching equations (5.1) with equations (3.4):

(5.2a)−αi(μi−qi∗)=−λaiαiQ(μi−qi∗)(5.2b)and −2αi(ai−Qαi)=−λaiαiQ(ai−Qαi).

To satisfy these equations, the variances $a_{i}$ must remain fixed at $Q/\alpha_{i}$. This forces all means to decay uniformly at a rate $\alpha_{i}\equiv \lambda$.

What does this geometric optimization mean for the actual thermodynamics of the network? The divergence $D_{1}^{*}$ is proportional to the excess free energy of the network relative to equilibrium [60,63]. Its time derivative is the negative of the excess entropy production [51,64]. So, when the system follows the gradient descent of $D_{1}^{*}$, it maximizes the rate at which this excess free energy is dissipated as heat into the surrounding medium [52,65], which corresponds to the excess entropy production. According to our result, to achieve the maximum dissipation rate (and greatest entropy production), the uncertainties in the expression of each gene must be held fixed, while all the means decay with identical rates. In information-theoretic terms, this corresponds to the fastest possible decrease of the inefficiency of describing the current state with a code optimized for equilibrium. Heuristically, the system *forgets* its initial condition the fastest [22].

**Figure 3.**
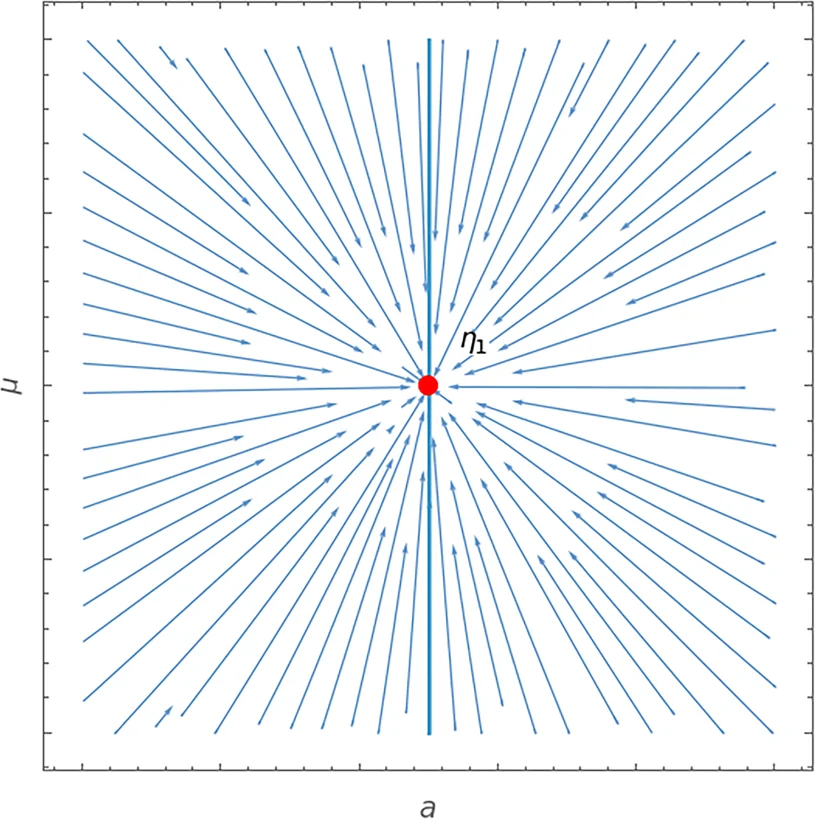
Gradient flow of $D_{1}^{*}$. The solid vertical line represents the trajectory of the only FP dynamics occuring along this flow.

## 6. Dynamics along geodesics

Viewing the dynamics of a GRN through the lens of information geometry reveals a key property about the paths that optimize information: they may be described as geodesic curves. To see this, let $\eta_{0}$ and $\eta_{1}$ denote the initial and final states of the gene network and consider the interpolation between them via an exponential mixture:

(6.1) $$p(q;t)=\frac{p(q;\eta_{1}{)}^{t}p(q;\eta_{0}{)}^{1-t}}{C(t)},$$

where C(t) is a normalization constant and 0≤t≤1. If this mixture lies within our parametric family (equation (3.2)), it traces a path in parameter space. To find this curve, denoted by η(t), take the logarithm and then differentiate with respect to t:

(6.2) $$\frac{\partial \ell_{q}}{\partial \eta^{i}}{\dot{\eta }}^{i}=\ell_{q}(\eta_{1})-\ell_{q}(\eta_{0})-\frac{d}{dt}\log C(t).$$

Notice that we used the chain rule on ${\dot{\ell }}_{q}$. We differentiate again and take expectations. This gives

(6.3) $$g_{ij}{\dot{\eta }}^{i}{\dot{\eta }}^{j}=\frac{d^{2}}{dt^{2}}\log C(t),$$

using $E_{\eta }[\partial_{i}\ell_{q}]=0$. This result relates the (Riemannian) speed of the curve to the normalization constant, telling us that log⁡C is a convex function. So, regardless of whether

(6.4) $$C(t)=\int_{Q}p(q;\eta_{1}{)}^{t}p(q;\eta_{0}{)}^{1-t}dq$$

increases or decreases, it will attain a minimum, whose negative is known as the *Chernoff distance*, between the initial and final distribution, used in hypothesis testing [22]. If in the previous step we multiply by $\partial_{k}\ell_{q}$ before taking expectations, we obtain

(6.5) $$g_{ki}{\ddot{\eta }}^{i}+E_{\eta }\left[\frac{\partial \ell_{q}}{\partial \eta^{k}}\frac{\partial^{2}\ell_{q}}{\partial \eta^{i}\partial \eta^{j}}\right]{\dot{\eta }}^{i}{\dot{\eta }}^{j}=0,$$

resorting to an alternative expression for the Fisher metrics: $g_{ij}=E_{\eta }[\partial_{i}\ell_{q}\partial_{j}\ell_{q}]$ [35].

The equation above represents a *geodesic*, a curve of constant velocity as measured by a *connection*. This geometric object embodies a parameter-independent notion of ‘straightness’, and is locally determined by a set of $(2N{)}^{3}$ functions known as *Christoffel symbols*. For more on connections, see appendix C.

Equation (6.5) means that, when the exponential interpolation of two distributions of the same family traces a curve on the parameter space, this curve is *straight* with respect to the so-called *exponential connection*, whose Christoffel symbols are

(6.6) $$\Gamma_{ij,k}^{*}:=E_{\eta }\left[\frac{\partial \ell_{q}}{\partial \eta^{k}}\frac{\partial^{2}\ell_{q}}{\partial \eta^{i}\partial \eta^{j}}\right].$$

Like the Fisher metrics, such a connection can be defined on the parameter space of any family of distributions. This is the second central object in information geometry.

By contrast to geodesics, relaxations to a steady state do not occur at constant speed, since equilibrium is reached asymptotically. If we allow for the speed of the geodesics above to decrease by changing the right-hand side to $-\lambda g_{ki}{\dot{\eta }}^{i}$, which amounts to a reparametrization of the curve, we find that equation (6.5) matches equations (5.1) (see appendix D). This tells us that the gradient descent of $D_{1}^{*}$ occurs along a *pregeodesic* of the exponential connection converging to $\eta_{1}$. In other words, relaxations maximizing the rate at which the system gains information from the steady state are exponential combinations of the initial and final states of the network.

For the divergence $D_{1}$, the analogous interpolation is linear: $p(q;t)=p(q;\eta_{0})(1-t)+p(q;\eta_{1})t$, with 0≤t≤1. Of course, the linear interpolation of two distributions of a given family, normal distributions in particular, does not necessarily belong to the same family, for all t. Let us assume for now that it does. Then, taking logarithms on both sides and differentiating yields

$$\begin{array}{r} \frac{\partial \ell_{q}}{\partial \eta^{i}}{\dot{\eta }}^{i}=\frac{p(q;\eta_{1})-p(q;\eta_{0})}{p(q;\eta_{0})(1-t)+p(q;\eta_{1})t}. \end{array}$$

If we differentiate again, we obtain

$$\begin{array}{r} \frac{\partial^{2}\ell_{q}}{\partial \eta^{i}\partial \eta^{j}}{\dot{\eta }}^{i}{\dot{\eta }}^{j}+\frac{\partial \ell_{q}}{\partial \eta^{i}}{\ddot{\eta }}^{i}=-{\left[\frac{p(q;\eta_{1})-p(q;\eta_{0})}{p(q;\eta_{0})(1-t)+p(q;\eta_{1})t}\right]}^{2}=-\frac{\partial \ell_{q}}{\partial \eta^{i}}\frac{\partial \ell_{q}}{\partial \eta^{j}}{\dot{\eta }}^{i}{\dot{\eta }}^{j}, \end{array}$$

with the last equality following from the previous equation. We do not get any relevant information if we take expectations on both sides. So, as we did before, we multiply by $\partial_{k}\ell_{q}$ and then take expectations, obtaining

(6.7) $$g_{ki}{\ddot{\eta }}^{i}+E_{\eta }\left[\frac{\partial \ell_{q}}{\partial \eta^{k}}\left(\frac{\partial \ell_{q}}{\partial \eta^{i}}\frac{\partial \ell_{q}}{\partial \eta^{j}}+\frac{\partial^{2}\ell_{q}}{\partial \eta^{i}\partial \eta^{j}}\right)\right]{\dot{\eta }}^{i}{\dot{\eta }}^{j}=0.$$

Like the case of exponential mixtures, the functions

(6.8) $$\Gamma_{ij,k}:=E_{\eta }\left[\frac{\partial \ell_{q}}{\partial \eta^{k}}\left(\frac{\partial \ell_{q}}{\partial \eta^{i}}\frac{\partial \ell_{q}}{\partial \eta^{j}}+\frac{\partial^{2}\ell_{q}}{\partial \eta^{i}\partial \eta^{j}}\right)\right]$$

define a connection, known as *mixture connection*. So, equation (6.7) says that when the linear interpolation of the initial and the final states of the network happens to be a normal distribution for all t, its mean and variance vary along a mixture geodesic.

Conversely, any mixture geodesic in parameter space corresponds to a linear interpolation of the endpoint distributions. Since the gradient descent of $D_{1}$ follows these curves at non-constant speed, the trajectories that maximize the rate at which the system sheds excess information are precisely convex combinations of the initial and final states.

**Figure 4.**
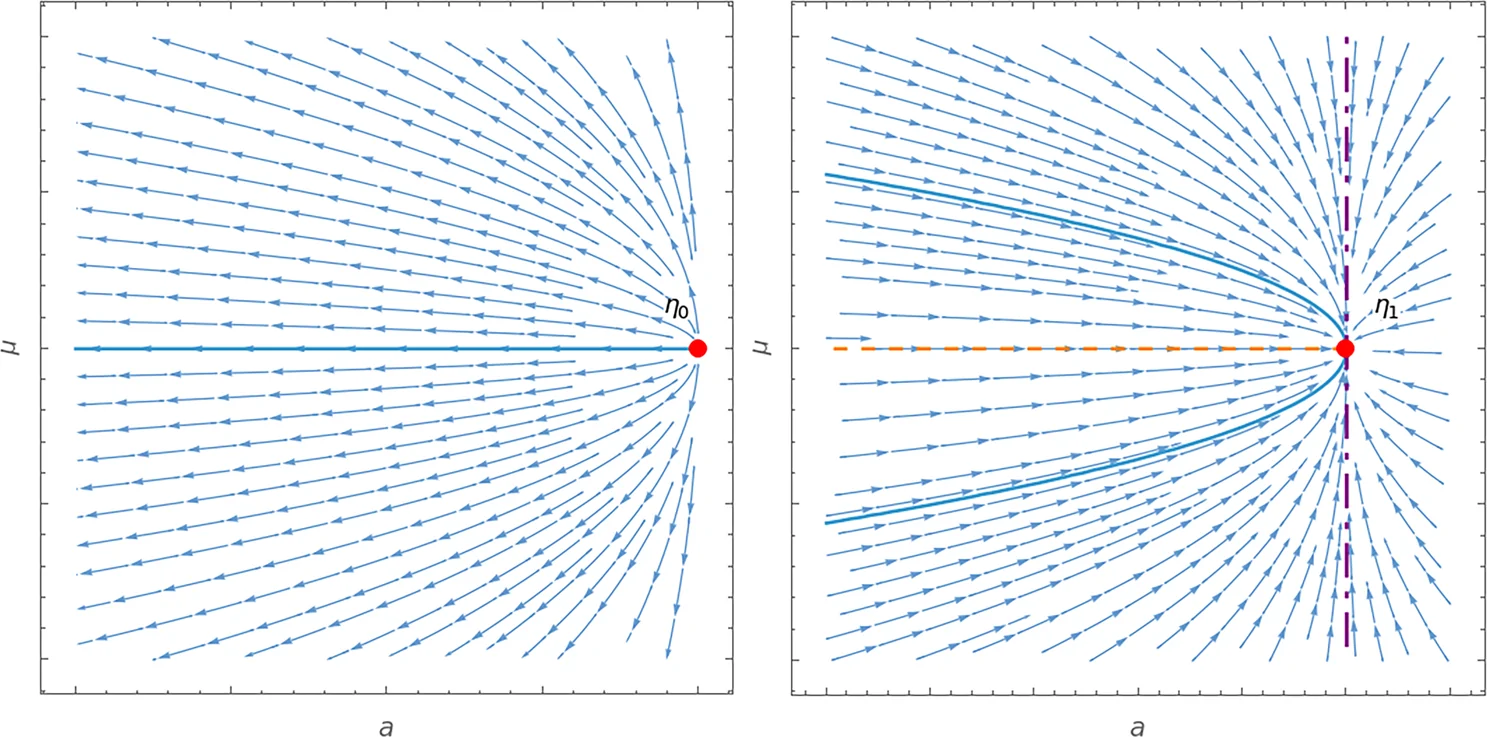
Left: flow of Fisher geodesics originating at $\eta_{0}$. Notice that the geodesics are ellipses. The solid, blue horizontal line is the only one corresponding to a trajectory of the FP dynamics. Right: as a graphical summary of our work, we show the FP dynamics converging to $\eta_{1}$ for one node. The solid curve is a mixture geodesic, the dot-dashed line is an exponential geodesic and the dashed line is a Fisher geodesic running in the opposite direction: it starts at $\eta_{1}$ and converges to a=0. The trajectories of these dynamics are parabolae: despite looking very similar to Fisher geodesics, they only coincide when they degenerate to a horizontal line.

The mixture connection is the third ingredient of information geometry. The relationship between this and the other two, the Fisher metrics and the exponential connection, is given by

$$\begin{array}{r} \partial_{k}g_{ij}=\Gamma_{ki,j}+\Gamma_{kj,i}^{*}. \end{array}$$

Any two connections satisfying this equation are called *dual*, and may be regarded as a generalization of the *Levi-Civita* (also *metric*) connection, which is uniquely determined by $\Gamma_{ki,j}^{g}=\Gamma_{ik,j}^{g}$ and

(6.9) $$\partial_{k}g_{ij}=\Gamma_{ki,j}^{g}+\Gamma_{kj,i}^{g}.$$

The Levi-Civita connection captures the concept of straightness naturally associated with the metrics through the inner product it defines at each point. Some juggling of equation (6.9) and the symmetry properties of the Christoffel symbols allow us to obtain them explicitly from the metric [58]:

(6.10) $$\Gamma_{ij,k}^{g}=\frac{1}{2}\left(\frac{\partial g_{jk}}{\partial \eta^{i}}+\frac{\partial g_{ik}}{\partial \eta^{j}}-\frac{\partial g_{ij}}{\partial \eta^{k}}\right).$$

Under certain conditions, metric geodesics define shortest paths in Riemannian geometry. So, it makes sense to ask if the dynamics in equations (3.4) ever minimizes the Fisher distance between the initial and the final state of the network. To answer to this question, we compare the geodesic equations for the Levi-Civita connection, which we compute in appendix D and illustrate in the left-hand panel of figure 4, with the relaxation dynamics on the parameter space. We do this by differentiating equations (3.4) and substsituting the result into the geodesic equations for the Levi-Civita connection. This yields

(6.11a)−αiμ˙i−μ˙ia˙iai=−λμ˙i(6.11b)and −2αia˙i+2μ˙i2−a˙i2ai=−λa˙i.

In this case, the only relevant dynamics takes place at constant means. This is because constant variances along a Fisher geodesic imply constant means, and non-constant $\mu_{i}$ and $a_{i}$ cannot occur along this flow since such dynamics requires that ${\dot{\mu }}_{i}^{2}=-\alpha_{i}a_{i}(0)$, which contradicts the fact that all the $a_{i}$ are positive. The unique Fisher geodesic that is also a trajectory of equation (2.1) is represented by a solid line on the left-hand panel of figure 4.

Furthermore, observe that if ${\dot{\mu }}_{i}=0$, then $\lambda -2\alpha_{i}-{\dot{a}}_{i}/a_{i}=0$. Solving for $a_{i}$ forces λ and Q to be both zero, comparing to equation (3.3*b*). Thus, only deterministic dynamics develops along Fisher geodesics. These paths approach the boundary $a_{i}\to 0$ asymptotically at constant speed: as the network evolves, the uncertainty about the expression level of each gene vanishes. Here, the dynamics lacks any biological significance, since noise, a fundamental ingredient of gene expression, is suppressed.

Kim [30] identifies Fisher geodesics as those that minimize the cumulative biological cost of changing the state of a system. So, according to our result, to minimize the biological cost of changing the state of a gene network, one must switch noise off, which results in undifferentiated states. In other words, some non-minimal biological cost is necessary for cell differentiation in the context of equation (2.1). We present a graphical summary of this section in the right-hand panel of figure 4. This figure shows the flow of the FP dynamics for one node of the network. The three superimposed curves are trajectories of this flow and geodesics at the same time. The solid curve corresponds to a mixture geodesic, the dot-dashed vertical line is an exponential geodesic, and the dashed horizontal line is a metric geodesic.

## 7. Concluding remarks and perspectives

Our informational study of the dynamics of GRNs provides interesting insights about these systems. When their evolution occurs along a geodesic, we can interpret the decay rates $\alpha_{i}$ as damping constants. Following this analogy, the most efficient dynamics, in terms of information, take place when this damping force is isotropic, i.e. all the $\alpha_{i}$ are the same. In this situation, noise and uncertainty in gene expression levels become the central characters in the two following scenarios.

First, we showed that the system optimizes informational efficiency, understood as shedding excess information in the fastest manner, as it evolves towards equilibrium by adjusting its uncertainties. When these are small, noise levels allow for a greater differentiation between initial and final states. Interpreting these trajectories from a thermodynamical perspective is an open question in the broader context of non-equilirium thermodynamics.

On the other hand, when the uncertainties in gene expression are kept constant, the dynamics maximizes the rate at which the system learns from the steady state. Here, the noise does not drive differentiation, but balances the decay rate to maintain these uncertainties at a fixed value. From the point of view of thermodynamics, the network releases excess free energy in the form of heat at the fastest possible rate, thereby maximizing the excess entropy production. We also showed that, in the regime where equation (2.1) holds, evolution with the minimum biological cost requires the suppression of noise. Here, the system evolves to a concentrated state with no uncertainty about the level of expression of its genes. This may be interpreted as noise driving cell fate determination, coinciding with similar existing interpretations [28,59,66] with experimental support [15,18].

The approach we adopted here also allows us to portray noise and decay rates as controls that can be tuned to drive the evolution of a network along a desired trajectory, which could be optimal in terms of information [67] or helpful in drug design [28,68].

From the point of view of biology, it is natural to wonder about the significance of the gradient descents of the KL divergences. It is well established that biological systems exploit noise for functional benefits, including probabilistic differentiation, signal detection and adaptation [15,18,59]. Moreover, selection is known to act on GRN architecture and on the variability of expression dynamics [69,70]. However, whether selection directly favours the maximization of information-theoretic rates remains an open question. Our results should rather be interpreted as providing a benchmark: if a system were under selective pressure for rapid information gain (or loss) during relaxation towards a fixed attractor (i.e. without altering the number or location of the attractors themselves), the optimal trajectories would be those we have identified. It is important to point out that, in more realistic scenarios, selection could act on the attractor landscape itself, which is beyond the scope of this work.

Our work does suggest directions for experimental testing. For instance, the prediction of coordinated, uniform decay rates across network nodes could, in principle, be tested by measuring mRNA and protein degradation rates during a well-characterized differentiation process, under conditions where cells are synchronously pushed towards a new attractor. To our knowledge, no experimental study has directly addressed predictions of this kind, but our framework provides a reference for what fastest information gain or loss during relaxation would look like, rather than a universal description of GRN dynamics.

The central role of noise in enabling efficient transitions also suggests an intriguing extension to population-level models. Our geometric framework could be used to explore whether a principle analogous to Fisher's fundamental theorem [71], where genetic diversity governs the rate of adaptive evolution, manifests in the context of cell populations navigating an epigenetic landscape. Specifically, one could investigate if the variance in gene expression states across a population predicts the efficiency of collective adaptation to new environmental attractors.

Another interesting direction for future work would be to model asymmetric relaxations in networks, where KL-equidistant initial states relax to equilibrium at different rates. This phenomenon is well documented in non-equilibrium systems [37] and has recently been addressed using the same information-geometric framework employed here [72]. Specifically, the role of the Amari–Chentsov tensor in predicting relaxation asymmetries could be explored within our model of GRN dynamics. This might reveal new principles underlying the fundamental differences between differentiation and reprogramming pathways.

The geometric framework we developed here is not restricted to the specific stochastic model of equation (2.1). Under certain conditions, the procedure of reducing stochastic dynamics to a deterministic, finite-dimensional one is quite general. Extending this approach to more general Markov processes is an active direction of our ongoing work.

Among the possible interpretations of our results, the thermodynamic one in terms of free-energy dissipation and excess entropy production stands out as the most firmly grounded, as it connects directly to the well-established gradient-flow structure of the FP equation. At this point, the informational language of ‘learning’ and ‘forgetting’ serves more as an heuristic to aid intuition.

Finally, we remark that we have considered only transitions to a fixed attraction basin, as a first approach to address the informational evolution of a network across the full epigenetic landscape from a geometric point of view in the future.

## Data Availability

This article has no additional data.

## Appendix A. Deterministic representation of stochastic dynamics and derivation of the Fisher metrics

This section provides detailed derivations and explicit calculations supporting the results in the main article. Equations (3.4) represent the time evolution of the transition probability of the states of the gene network and are obtained from equations (3.3) by differentiating

$$\begin{array}{r} {\dot{\mu }}_{i}=-\alpha_{i}(\mu_{i}(0)-q_{i}^{*})e^{-\alpha_{i}t}\text{ and}\;\;\;{\dot{a}}_{i}=-2\alpha_{i}\left(a_{i}(0)-\frac{Q}{\alpha_{i}}\right)e^{2\alpha_{i}t}, \end{array}$$

and then eliminating the parameter t. We do this solving for $e^{\alpha_{i}t}$ and $e^{-2\alpha_{i}t}$ in equations (3.3) and substituting into the equations above

$$\begin{array}{rl} {\dot{\mu }}_{i} & =-\alpha_{i}(\mu_{i}(0)-q_{i}^{*})\frac{\mu_{i}-q_{i}^{*}}{\mu_{i}(0)-q_{i}^{*}}=-\alpha_{i}\left(\mu_{i}-q_{i}^{*}\right)\\ \text{and }{\dot{a}}_{i} & =-2\alpha_{i}\left(a_{i}(0)-\frac{Q}{\alpha_{i}}\right)\frac{a_{i}-\frac{Q}{\alpha_{i}}}{a_{i}(0)-\frac{Q}{\alpha_{i}}}=-2\alpha_{i}\left(a_{i}-\frac{Q}{\alpha_{i}}\right). \end{array}$$

We assert that the parameter space spanned by $\left(\mu_{i},a_{i}\right)$ is a Riemannian manifold. Actually, the result is more general: the parameter space of *any* family of distributions has a metric tensor, intuitively, the ‘infinitesimal version’ of the KL divergence. Indeed, consider an expansion of $D_{1}$ to second order for $\eta =\eta_{1}+\delta \eta$. The first-order coefficient is

$$\begin{array}{rl} {\frac{\partial D_{1}}{\partial \eta^{j}}|}_{\eta =\eta_{1}} & ={\frac{\partial }{\partial \eta^{j}}|}_{\eta =\eta_{1}}\int_{Q}p_{1}\log\frac{p_{1}}{p_{\eta }}dq=-\int_{Q}{\frac{\partial p_{\eta }}{\partial \eta^{j}}|}_{\eta =\eta_{1}}dq=0. \end{array}$$

This means that changes in the parameter do not affect the KL divergence to first order. As usual, we are assuming that we can differentiate over the integral, and we denote p(⋅;η) by $p_{\eta }$. If we differentiate again, then

$$\begin{array}{rl} \frac{\partial^{2}D_{1}}{\partial \eta^{i}\partial \eta^{j}} & =-\frac{\partial }{\partial \eta^{i}}\int_{Q}\frac{p_{1}}{p_{\eta }}\frac{\partial p_{\eta }}{\partial \eta^{j}}dq=-\frac{\partial }{\partial \eta^{i}}\int_{Q}p_{1}\frac{\partial \ell_{q}}{\partial \eta^{j}}dq=-E_{1}\left[\frac{\partial^{2}\ell_{q}}{\partial \eta^{i}\partial \eta^{j}}\right]. \end{array}$$

The coefficients $g_{ij}(\eta_{1}):={\partial_{i}\partial_{j}D_{1}|}_{\eta =\eta_{1}}$ define a positive definite matrix, regardless of the values of the $\eta_{1}$ and of the family of distributions [35]. They are the components of the *Fisher metrics* defined on the parameter space.

We can also express $g_{ij}$ in terms of the covariance matrix of the $\partial_{j}\ell_{q}$ by differentiating the product $1/p_{\eta }\partial p_{\eta }/\partial \eta^{j}$ instead of grouping it as $\partial \ell_{q}/\partial \eta_{j}$:

$$\begin{array}{rl} {\frac{\partial^{2}D_{1}}{\partial \eta^{i}\partial \eta^{j}}|}_{\eta =\eta_{1}} & =-\int_{Q}p_{1}{\left(-\frac{1}{p_{\eta }^{2}}\frac{\partial p_{\eta }}{\partial \eta^{i}}\frac{\partial p_{\eta }}{\partial \eta^{j}}+\frac{1}{p_{\eta }}\frac{\partial^{2}p_{\eta }}{\partial \eta^{i}\partial \eta^{j}}\right)|}_{\eta =\eta_{1}}dq=E_{1}\left[{\left(\frac{\partial \ell_{q}}{\partial \eta^{i}}\frac{\partial \ell_{q}}{\partial \eta^{j}}\right)|}_{\eta =\eta_{1}}\right]. \end{array}$$

The second term inside the integral is zero because it is just the second derivative of $\int_{Q}p_{\eta }dq=1$ at $\eta =\eta_{1}$.

So, $D_{1}(\eta_{1}+\delta \eta )\approx \frac{1}{2}g_{ij}(\eta_{1})\delta \eta^{i}\delta \eta^{j}$, which shows that the Fisher metrics are the ‘infinitesimal version’ of the KL divergence.

In the particular case of the family defined by equation (3.2), we can write the Fisher metrics as the sum of the metric of each node of the network, provided that $\ell_{q}$ is a sum of logarithms of Gaussian distributions. In other words, we may write the Fisher metrics as a diagonal block matrix, each of which is the Fisher metrics of a normal family [35]:

$$\begin{array}{r} (g_{ij})=\left(\begin{array}{cc} 2/a & 0\\ 0 & 1/(2a^{2}) \end{array}\right). \end{array}$$

## Appendix B. Explicit calculations of gradient flows

Similarly, to obtain the KL divergence given by equation (4.1), we only need the KL divergence between two normal distributions [73]:

$$\begin{array}{r} D_{\text{KL}}(p(q;\mu_{1},a_{1})||p(q;\mu_{2},a_{2}))=\frac{1}{2}\left(\frac{a_{1}}{a_{2}}-\log\frac{a_{1}}{a_{2}}-1\right)+\frac{(\mu_{1}-\mu_{2}{)}^{2}}{a_{2}}. \end{array}$$

In what follows, we only do the explicit computations for one node.

We start with the gradient of the KL divergence. Recall that the gradient of a function f is the vector field associated with the differential of the function via the metric tensor: g(grad⁡f,⋅)=df, or in matrix notation, $\left(\mathrm{grad}f^{i}{)}^{T}(g_{ij})=(\partial_{j}f\right)$. The components of the gradient are thus obtained by multiplying $\left(\partial_{j}f\right)$ with the inverse of $\left(g_{ij}\right)$, this is, $(\mathrm{grad}f^{i})=[(\partial_{j}f)(g^{ij}){]}^{T}$. The gradient of

$$\begin{array}{r} D_{1}(\eta ):=D(\eta_{1}||\eta )=\frac{1}{2}\left(\frac{a_{1}}{a}-\log\frac{a_{1}}{a}-1\right)+\frac{(\mu_{1}-\mu {)}^{2}}{a} \end{array}$$

is then

$$\begin{array}{r} (\mathrm{grad}D_{1}^{i})=\left(\begin{array}{cc} a/2 & 0\\ 0 & 2a^{2} \end{array}\right)\left(\begin{array}{c} -2(\mu_{1}-\mu )/a\\ \left(a-a_{1}-2(\mu_{1}-\mu {)}^{2})/(2a^{2}\right) \end{array}\right)=\left(\begin{array}{c} \mu -\mu_{1}\\ a-a_{1}-2(\mu -\mu_{1}{)}^{2} \end{array}\right). \end{array}$$

The components of the gradient flow $\dot{\eta }=-\lambda \mathrm{grad}D_{1}$ are $\dot{\mu }=-\lambda (\mu -\mu_{1})$ and $\dot{a}=-\lambda [a-a_{1}-2(\mu -\mu_{1}{)}^{2}]$.

We follow a similar procedure for

$$\begin{array}{r} D_{1}^{*}(\eta ):=D(\eta ||\eta_{1})=\frac{1}{2}\left(\frac{a}{a_{1}}-\log\frac{a}{a_{1}}-1\right)+\frac{(\mu -\mu_{1}{)}^{2}}{a_{1}}, \end{array}$$

the gradient of which is

$$\begin{array}{r} (\mathrm{grad}{D_{1}^{*}}^{i})=\left(\begin{array}{cc} a/2 & 0\\ 0 & 2a^{2} \end{array}\right)\left(\begin{array}{c} 2(\mu -\mu_{1})/a_{1}\\ 1/2(1/a_{1}-1/a) \end{array}\right)=a/a_{1}\left(\begin{array}{c} \mu -\mu_{1}\\ a-a_{1} \end{array}\right). \end{array}$$

## Appendix C. Affine connections and their geodesics

There is a parameter-independent notion of straight lines, called *geodesics*. They are roughly defined as ‘straight’ trajectories, i.e. curves whose tangent vector is always *parallel* to the curve. So, defining geodesics relies on a definition of parallelism, realized by what is known as an *affine connection*.

Let us begin by explaining what affine connections are. Suppose that the vectors $\{e_{i}{\}}_{i=1}^{M}$ span all possible independent directions in which we can move starting from a point η in parameter space. These M vectors change from point to point, meaning that each $e_{i}$ is a vector field at each point η. Values of $e_{i}$ are always linearly independent, whence we call $\left\{e_{i}\right\}$ a *frame*.

Taking this into account, pick a vector $e_{i}$ and draw a curve that starts at η always tangent to $e_{i}$ as we move from point to point. After an infinitesimal displacement from η to η+δη, a vector $e_{j}$ might not have the same direction (relative to the curve) as it did at the beginning. ‘To first order’, intuitively speaking, the change in this vector will be $e_{j}^{\prime }=e_{j}+\Gamma_{ij}^{k}e_{k}$. The $\Gamma_{ij}^{k}$ are coefficients that measure how much we must correct on the kth direction to recover the original direction of $e_{j}$, as measured with respect to $e_{i}$. Clearly, when the $\Gamma_{ij}^{k}$ are all zero, the vector $e_{j}$ does not change along this curve, and we can say that $e_{j}$ is *parallel* to *itself* along the direction of $e_{i}$. Of course, the correction coefficients $\Gamma_{ij}^{k}$ might change from point to point, so they are functions of η.

The conclusion of this is that we may define a notion of parallelism by means of $M^{3}$ real functions of the parameters. This is called *affine connection*. The coefficients are the *Ricci rotation coefficients* of the connection, which are also called *Christoffel symbols* when each vector $e_{i}$ is tangent to the curve $\eta^{i}=\text{const.}$

What if we wish to determine when a vector $X=X^{j}e_{j}$ is parallel to itself along a curve tangent to $e_{i}$? In this case, we must also take into account the change in the components of X along the curve, $dX^{j}(e_{i})$. So, the total change of the vector as we move from η to η+δη is $dX^{j}(e_{i})e_{j}+X^{k}\Gamma_{ik}^{j}e_{j}$. This is what we call the *covariant derivative* of X with respect to the vector $e_{i}$ at η, which we denote by $(\nabla_{i}X{)}_{\eta }$. Usually, we refer to ∇ as *the connection* and to the $\Gamma_{ij}^{k}$ as its Ricci coefficients (or Christoffel symbols) relative to the frame $\left\{e_{i}\right\}$ (or to the parameters η).

We can extend covariant differentiation to arbitrary directions Y. We only need to add the contribution of each $\nabla_{i}X$ componentwise, i.e. $\nabla_{Y}X=Y^{j}\nabla_{j}X$.

Note that not any $M^{3}$ functions define a connection through the Christoffel symbols. For this, under a reparametrization η↦θ, the Christoffel symbols must change according to

$$\begin{array}{r} \Gamma_{ij}^{k}\mapsto {\Gamma^{\prime }}_{ij}^{k}=\frac{\partial \theta^{k}}{\partial \eta^{l}}\frac{\partial \eta^{m}}{\partial \theta^{i}}\frac{\partial \eta^{n}}{\partial \theta^{j}}\Gamma_{mn}^{l}+\frac{\partial \theta^{k}}{\partial \eta^{l}}\frac{\partial^{2}\eta^{l}}{\partial \theta^{i}\partial \theta^{j}}. \end{array}$$

It is not hard to see that the Christoffel symbols of the exponential and mixture connections transform correspondingly, and therefore define connections on parameter space.

Equipped with a connection, we can now pin down the concept of geodesic that we introduced heuristically before. We say that η(t) is a geodesic if its tangent vector does not change along the curve. In components, this is ${\ddot{\eta }}^{i}+\Gamma_{jk}^{i}{\dot{\eta }}^{j}{\dot{\eta }}^{k}=0$. Multiplying both sides by $g_{il}$ yields an equation in the form of equation (6.5), defining $\Gamma_{ij,k}:=g_{kl}\Gamma_{ij}^{l}$.

## Appendix D. The gradient descents of the Kullback–Leibler divergence are geodesics

Geodesics do not fully capture the idea of ‘straight line’. For instance, the curve $\left(x,y)=e^{t}(x_{0},y_{0}\right)$ is still a straight line in the Euclidean plane, since its tangent vector changes magnitude, but remains parallel to itself along the curve. This more general notion is that of a *pregeodesic*: a curve whose tangent vector does not change *direction*. In this case, the covariant derivative of $\dot{\eta }$ along $\dot{\eta }$ is not zero, but parallel to $\dot{\eta }$ itself, this is, $\nabla_{\dot{\eta }}\dot{\eta }=-\lambda \dot{\eta }$. Notice that λ is not necessarily constant.

In the particular case of normal distributions, the Christoffel symbols of the expontial connection are

$$\begin{array}{r} (\Gamma_{ij,1}^{*})=\left(\begin{array}{cc} 0 & -2/a^{2}\\ -2/a^{2} & 0 \end{array}\right)\text{ and}\;\;\;(\Gamma_{ij,2}^{*})=\left(\begin{array}{cc} 0 & 0\\ 0 & -1/a^{3} \end{array}\right). \end{array}$$

which, after some simplification, yield the pregeodesic equations

$$\begin{array}{r} \ddot{\mu }-\frac{2}{a}\dot{\mu }\dot{a}=-\lambda \dot{\mu }\;\text{and}\;\ddot{a}-\frac{2}{a}{\dot{a}}^{2}=-\lambda \dot{a}. \end{array}$$

To compare with the gradient descent of $D_{1}^{*}$, differentiate its components with respect to t:

$$\begin{array}{r} \ddot{\mu }=-\lambda \left[\frac{\dot{a}}{a_{1}}\left(\mu -\mu_{1}\right)+\frac{\alpha }{a_{1}}\dot{\mu }\right]\text{ and }\ddot{a}=-\lambda \left[\frac{\dot{a}}{a_{1}}(a-a_{1})-\frac{a\dot{a}}{a_{1}}\right]. \end{array}$$

Now, eliminate from the equations above $\mu_{1}$ and $a_{1}$ using equations (5.1):

$$\begin{array}{r} -\lambda \frac{\mu -\mu_{1}}{a_{1}}=\frac{\dot{\mu }}{a}\text{ and}-\lambda \frac{a}{a_{1}}=\frac{\dot{a}}{a}-\lambda . \end{array}$$

After substituting, this yields the pregeodesic equations for the exponential connection.

Let us turn our attention to the mixture connection. Its only non-zero Christoffel symbol is $\Gamma_{11,2}=2/a^{2}$. Then, the corresponding pregeodesic equations are

$$\begin{array}{r} \ddot{\mu }=-\lambda \dot{\mu }\;\text{and}\;\ddot{a}+4{\dot{\mu }}^{2}=-\lambda \dot{a}. \end{array}$$

To compare these with the gradient descent of $D_{1}$, differentiate equation (4.2*a*), which straightforwardly yields the first pregeodesic equation. For the a component, we differentiate equation (4.2*b*) with respect to t:

$$\begin{array}{r} \ddot{a}=-\lambda \dot{a}+4\lambda \left(\mu -\mu_{1}\right)\dot{\mu }. \end{array}$$

The desired equation follows after substituting equation (4.2*a*) to eliminate $\mu_{1}$.

Finally, to obtain the metric pregeodesics, we compute the Christoffel symbols of the Levi-Civita connection:

$$\begin{array}{r} (\Gamma_{ij,1}^{g})=\left(\begin{array}{cc} 0 & -1/a^{2}\\ -1/a^{2} & 0 \end{array}\right)\text{ and}\;\;\;(\Gamma_{ij,2}^{g})=\left(\begin{array}{cc} 1/a^{2} & 0\\ 0 & -1/(2a^{3}) \end{array}\right). \end{array}$$

Then, the metric pregeodesics are, after some simplification,

$$\begin{array}{r} \ddot{\mu }-\frac{\dot{\mu }\dot{a}}{a}=-\lambda \dot{\mu }\;\text{and}\;\ddot{a}+2{\dot{\mu }}^{2}-\frac{{\dot{a}}^{2}}{a}=-\lambda \dot{a}. \end{array}$$

To find the values of the parameters in the dynamics that yield metric geodesics, we differentiate equations (3.4) and substitute above. This yields equations (6.11), which we may rewrite as

$$\begin{array}{r} \dot{\mu }\left(\lambda -\alpha -\frac{\dot{a}}{a}\right)=0\;\text{and}\;\dot{a}\left(\lambda -2\alpha -\frac{\dot{a}}{a}\right)=-2{\dot{\mu }}^{2}. \end{array}$$

We claim that non-trivial dynamics occurs only when $\dot{\mu }=0$, assuming λ constant (so that the curve relaxes to a steady state as t→∞). If it were otherwise, we obtain from the first equation that $\dot{a}/a=\lambda -\alpha$. We may solve for a, obtaining $a=a_{0}e^{(\lambda -\alpha )t}$. Substituting this into the second equation yields $\alpha (\lambda -\alpha )a=2{\dot{\mu }}^{2}$, implying that (λ−α)<0. This would mean that a diverges as t→∞, which contradicts the fact that the network relaxes to a steady state. When the mean is kept constant, a similar procedure gives $a=a_{0}e^{(\lambda -2\alpha )t}$. We compare this to equation (3.3*b*), obtaining that λ=0 and Q=0.
